# Three-Layered Closure of Persistent Oroantral Fistula Using Chin Graft, Buccal Fat Pad, and Buccal Advancement Flap: A Case Report with Review of Literature

**DOI:** 10.1155/2019/8450749

**Published:** 2019-08-14

**Authors:** Shiv Prasad Sharma

**Affiliations:** Specialist Oral and Maxillofacial Surgeon, Zulfi General Hospital, Ministry of Health, King Abdulla Street, 11932, Saudi Arabia

## Abstract

Various techniques have been used for the repair of oroantral fistula (OAF) but majority of them have focused on the soft tissue closure alone, and most of the time, the osseous floor of the sinus was ignored. Existing literature supports that bone grafts supported by Buccal Fat Pad (BFP) heal well without undergoing significant resorption and necrosis. Through this case report, we wish to elaborate on the clinical success of using BFP and autogenous chin graft for simultaneous reconstruction of a large long-standing oroantral fistula with underlying osseous defect. The combination technique can prove beneficial for osseous regeneration of sinus floor and improve chances for future implant prosthetic rehabilitation.

## 1. Introduction

An oroantral fistula can develop as a sequel of dental extractions, infection, maxillary cyst/tumor excision, persistent infection/abscess, or radiotherapy [1, 2]. A small oroantral fistula < 3 mm can close spontaneously or by simple figure of eight suture, but large fistulae > 5 mm need more complicated surgical management [3–5]. It is shown that about 50% of patients with unattended OAF will develop maxillary sinus symptoms in 48 hours, and within 2 weeks, 90% will have maxillary sinusitis [6]. Early detection and management are advised to avoid further complications.

Management of established OAF can be classified into nonsurgical and surgical. Nonsurgical methods employ placing materials into the defect to act as a mechanical barrier without attempting flap closure. Synthetic graft materials, fibrin glue, xenograft, absorbable implants, and acrylic splints have all been used.

Surgical principle involves raising the adjacent or distant flap and advancing into the defect, for example, buccal flaps, palatal flaps, tongue flap, and nasolabial flaps [2]. All these methods have their own advantages and disadvantages.

The first clinical application of BFP was described by Egyedi in 1977 where he used it for reconstruction of palatal defect following tumor excision [7]. In recent years, Buccal Fat Pad (BFP) has been used successfully for closing oral defects due to its reliability and easy harvesting. With a proper technique of harvest, it can provide a 6 × 5 × 3 cm graft which can cover an area of 10 cm^2^. The mean thickness is about 6 mm [8, 9]. Care should be taken while harvesting to avoid injury to the parotid duct and facial nerve branches.

Ignoring the underlying bony defect can have serious consequences with respect to prosthetic rehabilitation. Simultaneous use of autogenous bone graft for OAF closure is recommended to facilitate good prosthetic treatment. Bone grafts from different sites have been used. In 1969, Proctor reported the use of iliac crest graft for large OAF closure [10]. This of course had the disadvantage of a separate surgical procedure associated with unacceptable donor site morbidities sometimes. The chin area, retromolar area, tuberosity, and ramus of mandible and zygomatic bone are the preferred alternative donor sites [11]. All the previous studies have utilized the standard buccal flap (Rehrmann) to cover the graft. We would like to present the successful use of autogenous chin graft covered with BFP and closure with BAF in the reconstruction of OAF.

The use of chin grafts in OAF closure has been reported with successful outcomes. Haas et al. in their preliminary study showed successful results with chin grafts [12]. It is important to note that although chin grafts have been used with successful results in numerous applications, their use in OAF closure has been scarce or may be under reported. It provides high quality bone which is easy to harvest and can be used without any significant donor site morbidity. The grafts from different origins show varied rates of mineralization. In an interesting study by Schlegel et al., chin bone grafts were found to be superior in terms of mineralization over a 6-month period [13].

Several techniques have been utilized to reconstruct the bony defect. Most of the published articles are in the form of a case report or case series. There is a lack of large prospective randomized studies comparing the reliability and outcomes of these existing methods. Kapustecki et al. in their series of 20 cases used autogenous grafts from symphysis mandible (14 patients) and external oblique line (6 patients) covered with PRF (Platelet Rich Fibrin) membrane [14]. They observed successful fistula closure along with restoration of adequate alveolar width and height in preparation for future prosthetic solutions. In their series of 21 cases reconstructed with bone grafts from mandibular symphysis and retromolar areas, Watzak et al. realized a complication rate of 14.3% in the form of wound dehiscence, which healed by secondary intention [15].

Er et al. performed two-layered closure with simultaneous bone grafting using graft from different intraoral sites [16]. They noted wound dehiscence in 20% of their patients. It can be realized that wound breakdown can result from inadequate two-layered closure. Ahmed and Askar showed favorable results with the coverage of bone graft with buccal mucoperiosteal flap [17]. In a 10-month follow-up case, Weinstock et al. demonstrated the additional benefit of the three-layer closure with bone graft, buccal fat, and buccal advancement flap [18]. The two layers over the graft not only provide added support but also give a well vascularized bed for the success of the graft.

Table 1 summarizes various studies demonstrating the use of different modalities addressing the osseous defect associated with OAF.

## 2. Case Presentation

**Figure 1:**
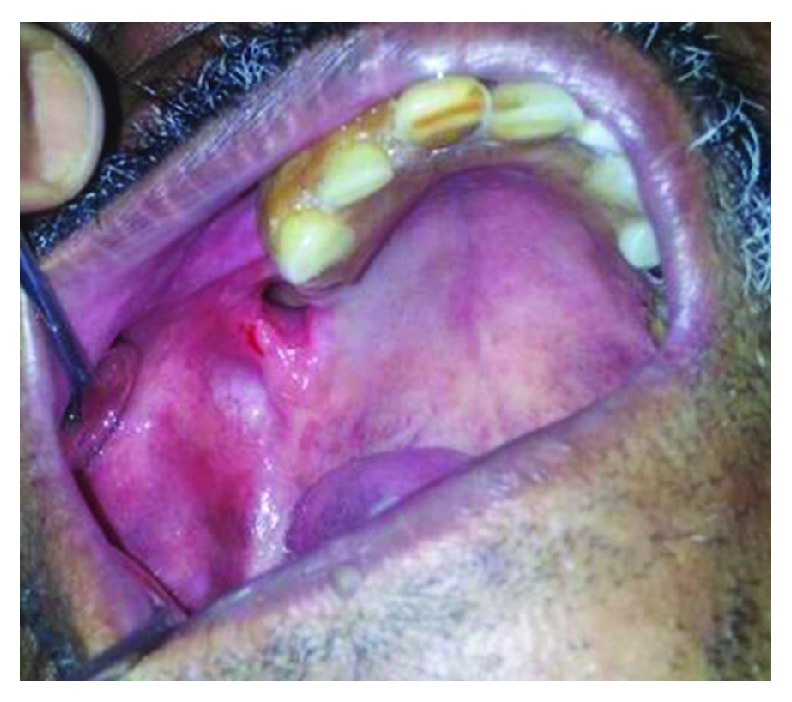
Clinical picture showing fistulous opening with identifiable bony defect.

A 45-year-old male patient, chronic smoker, reported to us with the chief complain of leakage of liquids through his nose while drinking. Otherwise, he was asymptomatic. He underwent extraction of his tooth number 16 about 6 months back elsewhere. On clinical examination, there were no signs and symptoms suggestive of acute maxillary sinusitis. Intraorally, a fistulous opening round in shape with normal surrounding mucosa and an obvious bony defect was seen along the maxillary alveolus molar region (Figure 1). No active discharge was present.

**Figure 2:**
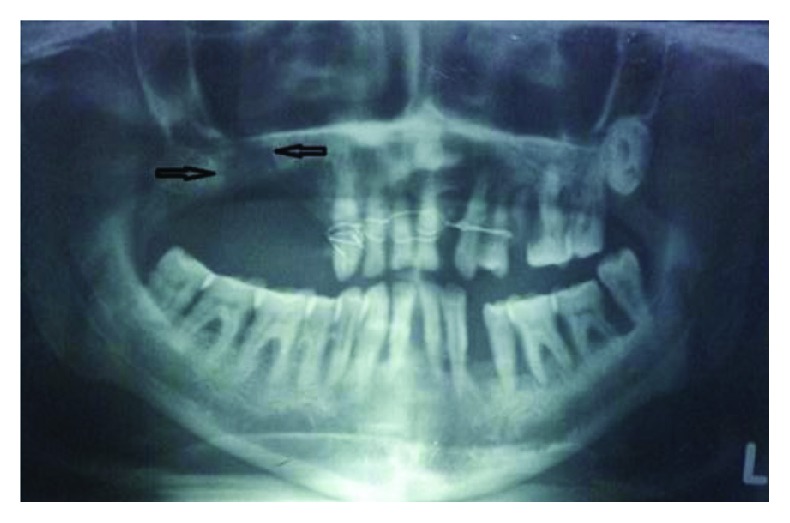
Panoramic X-ray demonstrating the underlying osseous defect.

Radiographic examination was done to define the underlying bony defect and also to rule out any foreign body (fractured root tip). The sinuses showed normal appearance. The bony defect of size 2 × 1.8 cm along the sinus floor was confirmed (Figure 2).

After correlating the clinical and radiographic findings, surgical closure using buccal advancement flap (BAF)+BFP+autogenous bone graft from the chin was planned. Preoperatively, the patient was started on antral regime (Tab amoxicillin clavulanic 625 mg+metronidazole 500 mg+Tab ibuprofen+Tab chlorpheniramine 4 mg + nasal decongestant) for 5 days.

**Figure 3:**
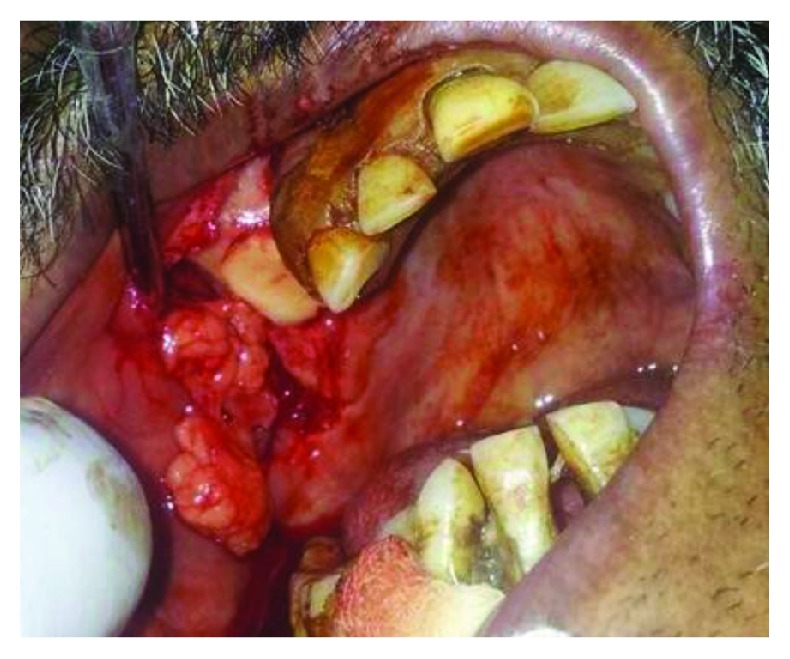
Harvested buccal fat+chin graft press fit into the bony defect.

**Figure 4:**
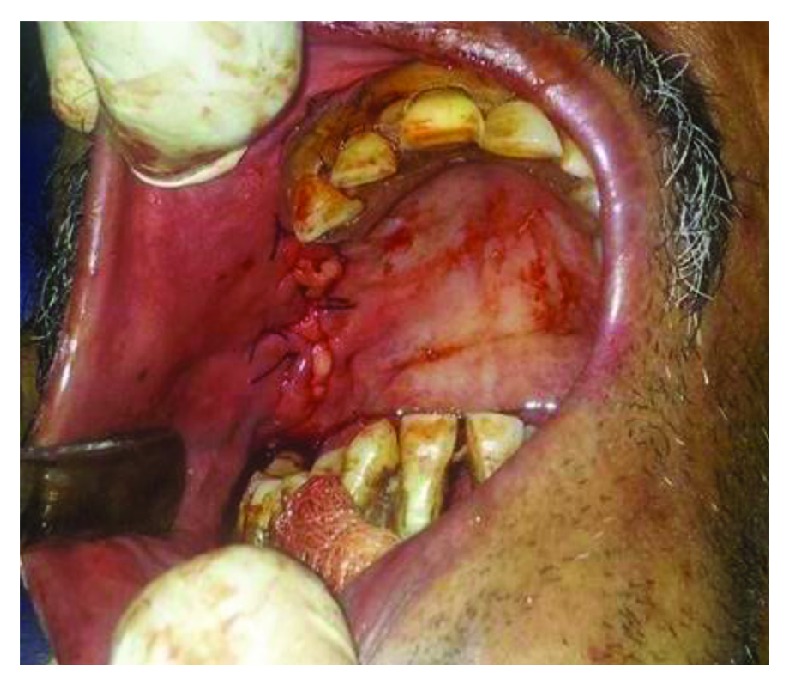
Three-layered closure achieved.

**Figure 5:**
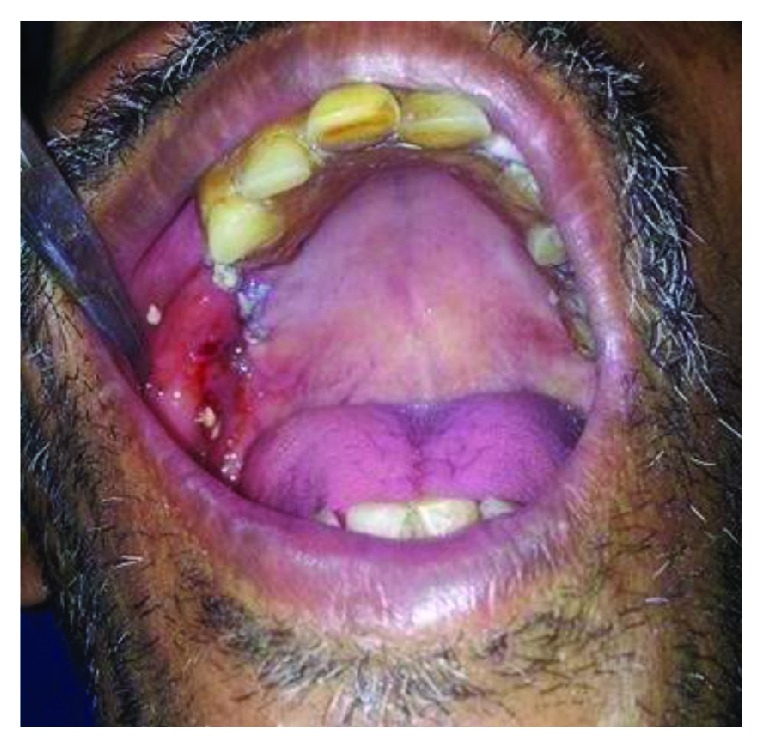
Mucosalisation of BFP noted at 1 week.

**Figure 6:**
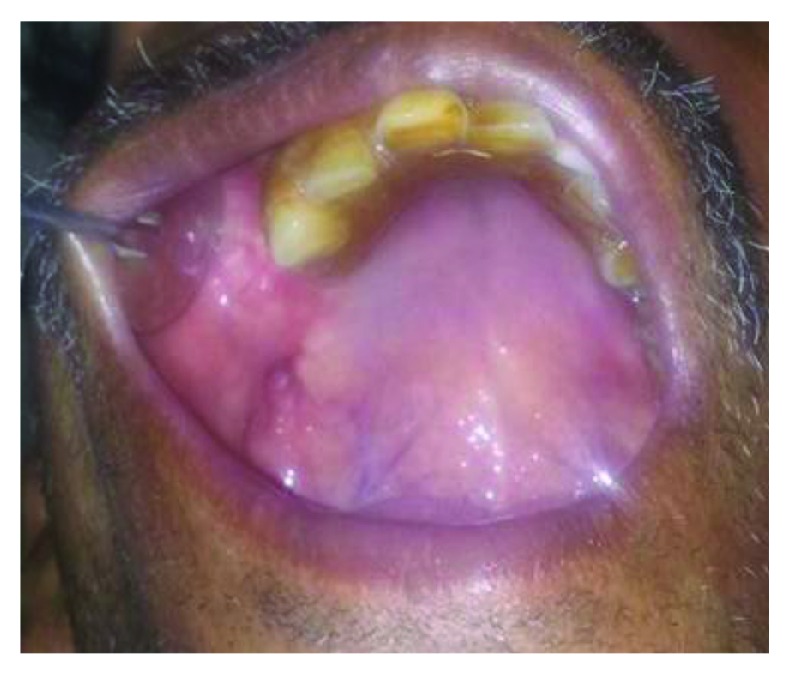
Complete healing after 1 month.

The entire procedure was performed under local anesthesia (2% lignocaine with 1 : 80000 adrenaline) using Posterior Superior Alveolar block supplemented by infiltration+greater palatine nerve block. The fistulous tract was excised in a circumscribed manner along the defect. A modified buccal mucoperiosteal flap was elevated in trapezoidal outline starting from the mesial and distal end of the fistulous opening. The periosteum was incised along the posterior aspect of the flap to identify BFP. Gentle blunt dissection was done to harvest the BFP till we obtained the sufficient amount of fat to cover the graft and the defect. Next, the cortical bone graft was harvested from the chin of size matching the defect. The graft was press fit into the defect and did not require screw fixation. Then, the BFP was mobilized to cover the graft, and finally, the mucoperiosteal flap was repositioned and sutured in place (Figures 3 and 4). This way, 3-layered closures were performed to reconstruct the bony as well as soft tissue defect. The patient was put on nasogastric tube feed till the time of suture removal. Mucosalisation of the fat pad was noticed at follow-up visits after 1 week and 1 month later (Figures 5 and 6).

**Figure 7:**
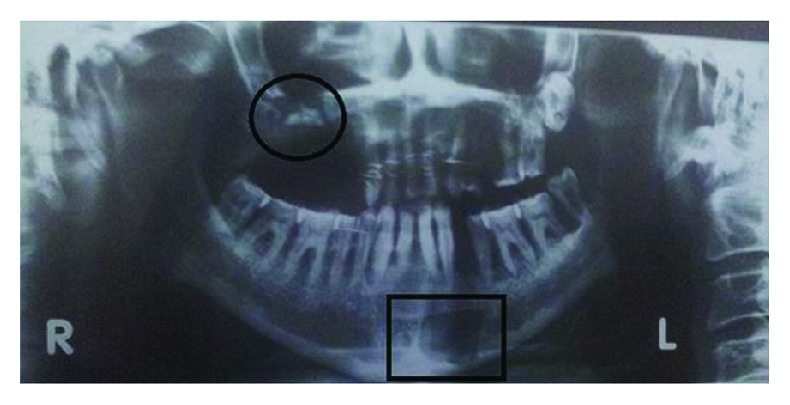
Radiograph showing chin graft well in place.

The patient was observed for signs of infection, dehiscence, and necrosis. Postoperative radiograph showed bone graft well in place (Figure 7). No significant complication was noted in the follow-up appointments. Both the primary site and the bone graft donor site healed without any dehiscence.

## 3. Discussion

The commonest cause for the development of OAF includes dental extractions of posterior teeth (molars, premolars) due to close proximity of the root apices to the sinus floor. Frequencies of such occurrences have been reported to be between 0.31% and 4.7% [19]. Based on its location, the fistula can be alveolosinusal, palatosinusal, or vestibulosinusal [20].

Ever since its first clinical use by Egyedi in 1977, BFP has gained popularity and rightly so because of its advantages: rapid epithelialization, excellent reliable blood supply, anatomically favorable position, ease of harvest, low rate of complications, and minimal to no donor site morbidity.

Problems that we can come across mainly at the time of harvesting BFP are perforation or shrinkage. Egyedi advocated covering the fat pad with split skin graft to overcome these issues, but Tideman et al. have shown that BFP was capable of self-epithelialization within 3-4 weeks of its inset [7, 21]. Nevertheless, covering the BFP might be essential in cases of large defects and where the amount of BFP may be inadequate. In such cases, buccal advancement flap is the best option. This combination technique provides more stability and provides additional tissue for cover. In our case, this layered closure was needed to provide support to the bone graft and also to maintain a well vascularized environment for the graft take up.

Our idea of three-layer closure is supported by the studies of Er et al. and Weinstock et al. [16, 18]. Er et al. observed wound dehiscence in 20% of cases after two-layered closure. Weinstock et al. demonstrated additional benefit of the buccal flap covering the BFP over the graft in a study with a 10-month follow-up.

There is so much heterogeneity in the methods for OAF closure, and no particular technique is superior or inferior to other. Each case is at the discretion of the treating surgeon based on his experience and preference. The choice of the technique must be guided by 4 important assessment criteria: (a) size and type of defect, (b) presence or absence of sinus disease, (c) minimal donor site morbidity to the patient, and (d) prosthetic considerations. The described technique has the added advantage for graft support and minimizing the chance of graft resorption or wound dehiscence.

## 4. Conclusion

Repair of OAF is essential to prevent further complications related to the maxillary sinus. The routine flap based techniques will not be sufficient alone due to the continued demand and necessity for implant rehabilitation. Due consideration should be given to the underlying osseous defect as well.

Therefore, we used autogenous bone graft harvested from the chin for the closure of bone. BFP and chin graft may be adequate to cover a large defect, but BAF may serve as an additional support and will help in minimizing any complication related to the grafts.

## Figures and Tables

**Table 1:** Summarizes various studies demonstrating the use of different modalities for OAF closure.

Author/year	Number of cases	Type of bone graft
(1) Waldrop TC et al. (1993)	1	Gelatin membrane+DFDBA+ePTFE
(2) Liversedge et al. (2002)	1	BFP+maxillary bone graft
(3) Haas et al. (2003) [12]	5	Chin graft+buccal flap
(4) Watzack G et al. (2005)	21	Retromolar/chin graft
(5) Delgado Galindez (2005)	22	Mandibular graft+mucoperiosteal flap
(6) Ogunsalu et al. (2005)	1	Biooss sandwiched between Biogide
(7) Scala M et al. (2007) bovine bone	3	Cryoplatelet gel+particulate bone maxilla+
(8) Penarrocha Diago M et al. (2007)	1	Zygomatic bone
(9) Lee BK (2008)	1	Ileac bone+palatal flap
(10) Doobrow JH et al. (2008)	1	Collagen matrix+FDB+CaSO_4_
(11) Scattarella et al. (2010)	1	Autologous bone+particulate xenograft+PTFE
(12) Ahmed MS et al. (2011)	8	Chin/ramus graft+BAF
(13) Er et al. (2013) [16] wall (3)	10	Chin (3), buccal (1), tuberosity (2), ramus (1), maxillary
(14) Cottam JR et al. (2013)	1	Recombinant rhBMP-2+collagen matrix
(15) Pourdanesh F et al. (2013)	1	BFP+pedicled coronoid process+mucosa
(16) Weinstock RJ et al. (2014) [18]	1	Maxilla+BFP flap+buccal advancement flap
(17) De Biasi M et al. (2014)	20	BFP+hydroxyapatite crystals+collagen sheath
(18) Choi N et al. (2015)	1	Scapular tip free flap
(19) Kapustecki M et al. (2016) [14]	20	Mental protuberance (14)+oblique line (6)
(20) S Amroun et al. (2018)	1	Maxillary tuberosity+mucosal closure

Abbreviations: DFDBA: Decalcified Freeze-Dried Bone Allograft; ePTFE: extended polytetrafluoroethylene; BFP: Buccal Fat Pad; Biooss: bone graft material; Biogide: a resorbable membrane; FDB: Freeze-Dried Bone; CaSO_4_: Calcium Sulphate; BAF: buccal advancement flap; rhBMP-2: recombinant human Bone Morphogenetic Protein-2.
